# Does the combination of resistance training and a nutritional intervention have a synergic effect on muscle mass, strength, and physical function in older adults? A systematic review and meta-analysis

**DOI:** 10.1186/s12877-021-02491-5

**Published:** 2021-11-12

**Authors:** MoonKi Choi, Hayeon Kim, Juyeon Bae

**Affiliations:** 1 College of Nursing, Kangwon National University, Chuncheon-si, Gangwon-do Republic of Korea 24341; 2 Seoul Women’s College of Nursing, Ganhodae-ro 38, Seodaemun-gu, Seoul, Republic of Korea 03617; 3 Department of Nursing, Yeoju Institute of Technology, Sejong-ro 338, Yeoju-si, Gyeonggi-do Republic of Korea 12652

**Keywords:** Exercise, Frailty, Nutrition, Older adults, Resistance training, Sarcopenia

## Abstract

**Background:**

Health-promoting interventions are important for preventing frailty and sarcopenia in older adults. However, there is limited evidence that nutritional interventions yield additional effects when combined with resistance training. This systematic review and meta-analysis aimed to compare the effectiveness of nutritional interventions with resistance training and that of resistance training alone.

**Methods:**

Randomized controlled trials published in peer-reviewed journals prior to July 2020 were retrieved from databases and other sources. The articles were screened according to the inclusion and exclusion criteria. The methodological quality of the included studies was assessed using Cochrane’s risk of bias tool 2. A meta-analysis was performed using the RevMan 5.4 program and STATA 16 program.

**Results:**

A total of 22 studies were included in the meta-analysis. The results of the meta-analysis showed no significant differences between groups in muscle mass, muscle strength, or physical functional performance. In the subgroup analysis regarding the types of nutritional interventions, creatine showed significant effects on lean body mass (*n* = 4, MD 2.61, 95% CI 0.51 to 4.72). Regarding the other subgroup analyses, there were no significant differences in appendicular skeletal muscle mass (*p* = .43), hand grip strength (*p* = .73), knee extension strength (*p* = .09), chair stand test results (*p* = .31), or timed up-and-go test results (*p* = .31). In the meta-regression, moderators such as the mean age of subjects and duration of interventions were not associated with outcome variables.

**Conclusions:**

This meta-analysis showed that nutritional interventions with resistance training have no additional effect on body composition, muscle strength, or physical function. Only creatine showed synergistic effects with resistance training on muscle mass.

**Trial registration:**

CRD42021224843.

**Supplementary Information:**

The online version contains supplementary material available at 10.1186/s12877-021-02491-5.

## Background

Age-related conditions and chronic diseases increase the risk of disability and dependence, which are considered nearly irreversible conditions. Increasingly more older adults are becoming interested in ‘active aging’, which refers to the process of optimizing opportunities for health, participation, and security later in life [1]. A growing research topic is the identification of factors that increase the risk of negative events and the development of preventive interventions against disability. In this context, frailty and sarcopenia have increasingly emerged as research interests.

Although there is still no consensus on the definition and measurement of frailty for diagnosis, frailty is defined as a geriatric condition characterized by a cumulative decline in functioning and accompanied by increased vulnerability to stressors and dependency [2]. In 2001, Fried et al. [3] suggested the following criteria of frailty as a physical phenotype, focusing on physiological components: unintentional weight loss, exhaustion, decreased physical activity, a slow walking speed, and muscle weakness. Rockwood and Mitnitski [4] introduced a frailty index based on the accumulation of age-related deficits. A recent consensus more broadly suggested that frailty is a multidimensional syndrome including sensory limitations, cognitive decline, mood-related conditions, changes in the social environment, comorbidities and disability in addition to physical impairment [5]. The specific pathological pathway of frailty remains unclear, but frailty has a biological component resulting from inflammation and cumulative cellular damage over one’s lifetime. Although it occurs independently of chronological age, frailty is more prevalent in people of an older age; females; those who are living alone; those with low educational and socioeconomic statuses, multimorbidity, malnourishment, depression, polypharmacy, cognitive impairment, and a low physical activity level; and those who smoke and drink alcohol regularly [6–8].

Sarcopenia is considered a muscle disorder associated with poor muscle function; low muscle mass is considered a principal determinant. Although sarcopenia occurs in people who are not elderly, muscle mass decreases with age [9]. There are several operational definitions of sarcopenia; for example, the European working group on sarcopenia in older people defines sarcopenia as a combination of low muscle mass and strength and/or poor physical function [10]. Inconsistency in the definition leads to a wide range of prevalence rates, ranging from 9.9 to 40.4% [11]. Although the concepts of both frailty and sarcopenia are still being developed, the physical phenotypes of frailty, including low grip strength and slow gait speed described by Fried et al. [3], overlap substantially with those of sarcopenia [12]. In addition, as the etiology of frailty, such as inflammation, cellular damage, and protein degradation, is also related to that of sarcopenia, sarcopenia is an essential component of physical frailty. Frailty with sarcopenia can result in falls and fractures, a loss of independence, disability, morbidities, social isolation, institutionalization, and hospitalization [6, 13, 14], which lead to increases in healthcare costs and social burden [15]. Physical frailty and sarcopenia are transitional processes that increase individuals’ vulnerability to reduced functional capacity and adverse health outcomes. Issues related to healthcare and support for frail and sarcopenic older adults are expected to increase with population aging [16].

Health-promoting behaviors are important to prevent disability and dependence and to reduce the need for care [17]. Physical inactivity and malnutrition are common conditions in older adults and are major modifiable risk factors for frailty and sarcopenia [18, 19]. An increasing amount of research has suggested that physical inactivity can lead to the loss of muscle mass, decreases in muscle strength and poor physical performance. Several evidence-based systematic reviews and meta-analyses of RCTs have shown that exercise affects muscle mass, strength, and physical performance [17, 20]. For optimal effects, multimodal exercise combined with moderate- to high-intensity progressive resistance training and functional balance and mobility training at least twice a week for 30–45 min per session is recommended [19, 21].

Several nutrients, such as protein and vitamins D and E, have been known to affect anabolic stimuli, lead to the synthesis of muscle proteins, and protect against oxidative damage and the loss of muscle mass [22]. Although nutrition plays a key role in the pathogenesis of physical frailty and sarcopenia, the effects of nutritional interventions on muscle mass, strength and physical function are unclear. A systematic review showed that dietary supplementation, when combined with exercise training, has been shown to yield additional effects on muscle mass, strength and physical performance in some studies, but the existing evidence was inconsistent [23]. A more recent systematic review and meta-analysis by Hita-Contreras et al. showed that nutritional interventions do not provide additional or synergistic benefits when combined with resistance exercise in terms of muscle strength and mobility improvements among older adults with sarcopenic obesity [20].

There is evidence suggesting that there is an interaction effect between exercise and various nutritional factors, particularly protein and some multinutrient supplements, that can slow age-related decline and preserve muscle function in older adults. However, whether this has a meaningful preventive effect on frailty and sarcopenia remains unclear. Some previous reviews did not provide a quantitative synthesis, combined community-dwelling and institutionalized populations, or included and analyzed diverse types of interventions together [17, 23, 24], making it difficult to interpret the results. Thus, we focused on the primary prevention and synergistic effects of nutritional interventions, that is, the changes in muscle function after resistance training and nutritional interventions, in healthy community-dwelling older adults. The aim of this systematic review and meta-analysis was to compare the combination of resistance training and nutritional interventions with resistance training alone. A preferred reporting items for systematic reviews and meta-Analyses (PRISMA) checklist is presented in Additional file 1.

## Methods

### Search strategy

Electronic databases and the reference lists of related studies were searched by two investigators. First, for the electronic search, MEDLINE (PubMed), Cochrane CENTRAL, and Embase were searched for articles published prior to July 2020 by entering the following combinations of keywords: (“nutrition” OR “food” OR “diet”) OR (“exercise” OR “resistance training”) AND “aged” AND (“muscle mass” or “skeletal muscle” OR “muscle strength” OR “physical performance” OR “physical functional performance” OR “walking speed” OR “gait speed”). Second, the reference lists of related studies were searched to identify additional articles. The searches were limited to articles published in the English language, studies involving humans, and RCTs. Only peer-reviewed articles were included, and gray literature such as dissertations, proceedings, and government reports was excluded.

### Study selection

The inclusion criteria for this systematic review were as follows: (a) studies including community-dwelling healthy older adults aged 60 years or above; (b) those including experimental groups that underwent resistance training and nutritional interventions; (c) those including comparison groups that underwent resistance training alone with or without a nutritional placebo supplement; (d) studies that reported the outcome measures of muscle mass, muscle strength, and physical functional performance; and (e) randomized controlled parallel-group trials with at least one arm. We set the age of 60 years or above, the age at which the activity level can decrease after retirement [25] and the loss of muscle strength accelerates [26]. We included only studies in healthy subjects to reduce the level of heterogeneity between studies. We accepted the various authors’ own definitions of ‘healthy’. The experimental interventions included any form of resistance training and nutritional (dietary) interventions that involved repeated practice during standardized programs for the purpose of enhancing muscle mass, muscle strength, and physical function. Nutritional interventions were defined as those that provided at least one nutrient through nutritional supplementation or whole food to obtain biologically beneficial effects. There was no minimum duration of follow-up. However, all included trials had to report outcomes at a minimum of one time point after the completion of the intervention.

Articles were excluded if (a) the participants had malignant tumors, severe chronic diseases, or levels of frailty and sarcopenia that limited their physical activity, diet, and level of independence in daily life; (b) the study was conducted in an animal model; (c) the experimental intervention was combined with any other form of interventions, such as medication and hormone therapy; (d) the nutritional intervention was designed for calorie intake reduction and weight loss; (e) the study evaluated the effectiveness of experimental interventions by only examining inflammatory factors or biological markers related to muscle synthesis; or (f) the study had a non-RCT design, such as case reports or cohort studies, without a comparison group.

Studies were selected based on the inclusion and exclusion criteria by two independent researchers; these researchers screened the studies according to the titles and abstracts of all studies and then reviewed the full texts of the remaining studies. Disagreements between researchers were resolved by discussion.

### Data extraction

Two independent researchers extracted key data from the included articles in a standardized Excel sheet, and the results were cross checked. For each article, data about (a) the article, including the authors, year of publication, and country; (b) characteristics of the study population, including the number of participants, mean age, sex, health status, and attrition rate; (c) characteristics of the experimental intervention, including the contents of resistance training, contents of nutritional intervention, delivery mode, amount, frequency and duration of intervention, and treatment for comparison group; and (d) outcome evaluation, including the follow-up period and the method of measurement. As the aim of the study was to compare the effects of the combination of resistance training and nutritional interventions with those of resistance training alone on muscle mass, strength, and physical performance, when more than two groups were present, only the data we intended to compare were recorded.

### Assessment of risk of Bias

Methodological quality was assessed using Cochrane’s risk of bias 2 (RoB2) tool by two independent researchers. The RoB2 tool consists of five domains: the randomization process, deviation from intended intervention, missing outcome data, measurement of outcome, and selection of the reported result. The risk of bias for each domain is evaluated as “low risk”, “some concerns”, or “high risk” by an algorithm with several signaling questions. Overall, “low risk of bias” was recorded when the study was judged to have a low risk of bias for all domains, “some concerns” was recorded when the study was judged to have some concerns in at least one domain, and “high risk of bias” was recorded when the study was judged to have a high risk of bias in at least one domain. This process was carried out by two independent researchers, and inconsistencies were resolved through discussion.

### Data synthesis and statistical analysis

The effect sizes of the combination of resistance training and a nutritional intervention were calculated using the mean difference (MD) or standardized MD (SMD) for continuous outcome data for muscle mass, muscle strength, and physical functional performance. When a study provided data on more than one outcome for the same construct (ex: timed up-and-go and 4-m walk tests for physical functional performance), valid, reliable and commonly used measures for frailty and sarcopenia were selected by reviewing the associated literature and considering the frequencies of their use in the included studies. As a result, lean body mass and appendicular skeletal muscle mass were selected for muscle mass, hand grip strength and knee extension strength for muscle strength, and the chair stand tests and timed up-and-go tests for physical functional performance. Fat-free mass was included in the analysis when lean body mass was not available.

In addition, if a study used different lengths of intervention and follow-up periods, we used the outcome values at the postintervention endpoint. When only the mean change scores and standard deviation (SD) of each group were available, they were used instead of the postintervention endpoint mean and SD for the mean difference. SMDs were used for studies using different units (scale) of the same measure (ex: kg and Nm for strength). If there were more than two groups that could be considered experimental groups in the study, the groups were combined to create a single pairwise comparison in the meta-analysis to avoid unit-of-analysis error from multiple comparisons as recommended [27]. Studies for which we could not identify the outcome data necessary for quantitative synthesis after contacting the authors were excluded from this meta-analysis.

Meta-analysis was conducted using Review Manager (RevMan) 5.4. Individual MDs and SMDs were pooled using random effects models and the inverse variance method. The statistical significance of each effect size and overall effect size were checked using 95% confidence intervals. The chi-squared test and Higgin’s I^2^ test were used to examine between-trial heterogeneity. When the *p value* for the chi-squared test was less than 0.1 and I^2^ was greater than 50%, substantial heterogeneity was considered to be present. Subgroup analysis was conducted by nutritional intervention type. All subgroup differences were tested regarding the significance of the effect sizes and heterogeneity. Meta-regression was conducted to identify the potential effect moderator using the STATA 16 program. *p value*s from random effect meta-regression were calculated using restricted maximum likelihood for continuous moderators.

### Certainty of evidence

The Grading of Recommendations Assessment, Development and Evaluation (GRADE) tool approach was used to assess the quality of evidence. It compared the resistance training combined diet compared to resistance training only for muscle function. The GRADE tool comprised of risk of bias, inconsistency, indirectness, imprecision, publication bias. The grading was estimated as high, moderate, low and very low certainty of quality.

## Results

### Search results

**Fig. 1:**
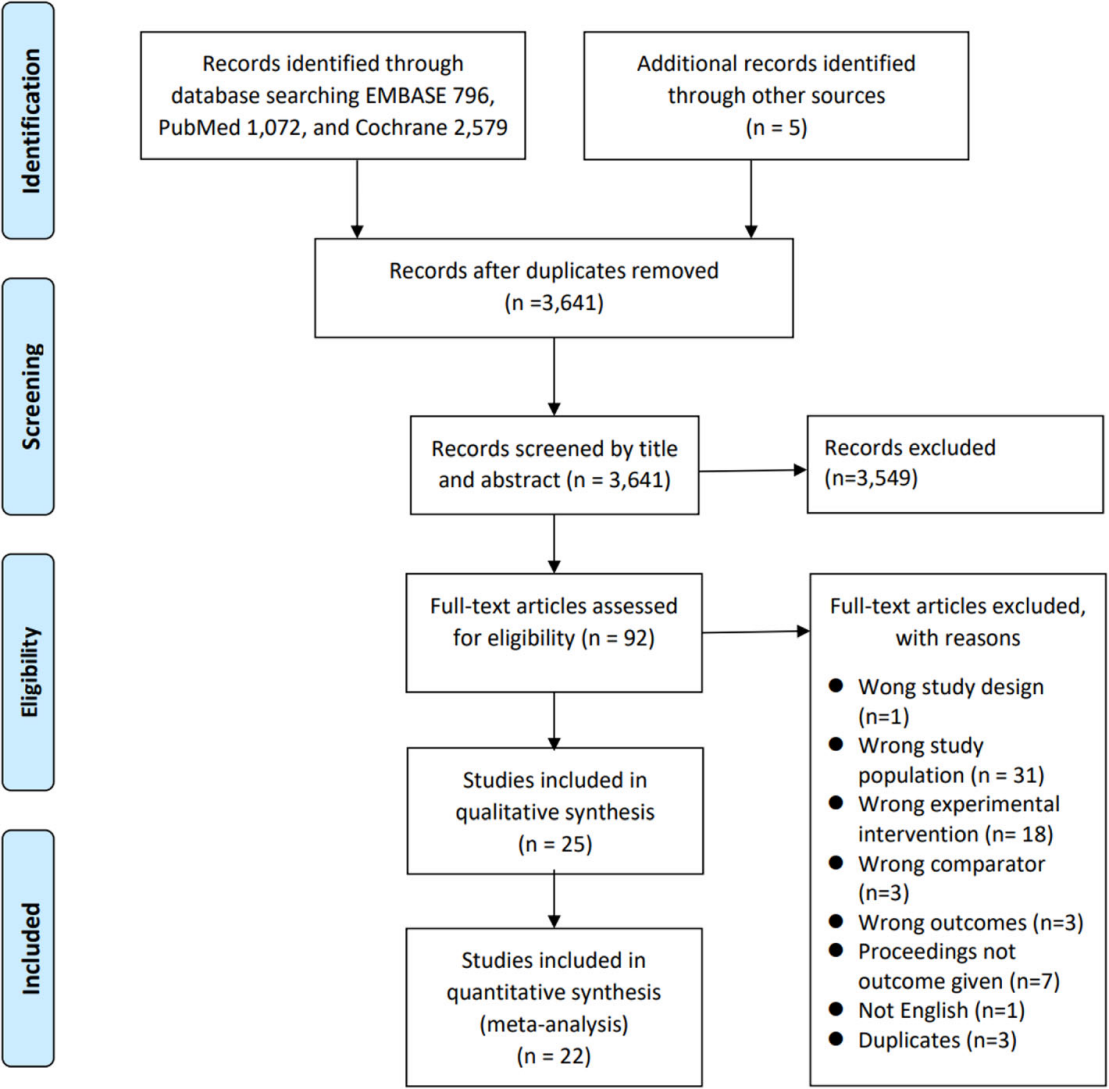
Flow diagram of the study selection process

Figure 1 demonstrates the study selection process. After duplicates were removed, 3641 articles remained. After 3549 articles were excluded through title and abstract review, the full texts of 92 articles were reviewed. Sixty-seven articles were additionally excluded, and consequently, 25 articles were included in this systematic review. In some papers, the necessary values for meta-analysis could not be identified, so 22 articles were included in the quantitative synthesis.

### Description of included studies

Table 1 shows the characteristics of the included studies. Six RCTs were conducted in Canada, six in Japan, four in Brazil, two in the Netherlands, two in the USA and one each in France, Iceland, Norway, Sweden, and the UK. The studies were published between 1998 and 2020. The sample sizes ranged from 14 to 161. Six studies were conducted in males only, five studies were conducted in females only, and 14 studies were conducted in both males and females. There were 12 studies with a mean age of participants of less than 70 years and 13 studies with a mean age of more than 70 years.

**Table 1:** Summary of included study characteristics

First author (year), study location	Sample characteristics: n, mean age ± SD, sex (female ratio)	Intervention group		Control group^a^	Follow-up period (weeks)^b^	Body composition assessment method	Muscle strength assessment method	Physical performance assessment method
		Exercise	Nutrition					
Aguiar (2013) [28], Brazil	I^c^: 9, 64 ± 4, female C^d^: 9, 65 ± 6, female	Resistance training using machines, 60 min sessions, 3 times a week, for 24 weeks	5 g Creatine monohydrate, once a day, for 12 weeks	Placebo: maltodextrin	24	Body mass Fat free mass Muscle mass	Bench press Biceps curl Knee extension	Chair stand
Aoki (2018) [29], Japan	I: 43, 68.8 ± 5.3, mixed (74.4%) C: 45, 71.2 ± 6.8, mixed (75.6%)	Lower body resistance training, daily	25mcg vitamin D3 (1000 IU^e^), divided into 3 times, daily	Usual diet	24	BMI^f^ Lower limb muscle mass	Hip flexion Knee extension	Chair stand Single leg stance Two step test Functional reach test
Arnarson (2013) [30], Iceland	I: 83, 73.3 ± 6, mixed (*unknown*) C: 78, 74.6 ± 5.8, mixed (*unknown*)	Resistance training using machines, 3 times a week	20 g of whey protein 3 times a week	Placebo: 250 ml isocaloric carbohydrate drink	12	ASMM^g^ Lean body mass	Grip strength Knee extension	TUG^h^ 6-min walk
Bermon (1998) [31], France	I: 8, 71.0 ± 5.4, mixed (50%) C: 8, 69.3 ± 1.1, mixed (50%)	Resistance training using machines, 3 times a week, for 7 weeks	First 5 days: 5 g creatine monohydrate and 2 g glucose, 4 times a day Following 47 days: 3 g creatine monohydrate and 2 g glucose once a day	Placebo: First 5 days:7 g glucose 4 times a day Following 47 days: 5 g glucose once a day	8	Body mass BMI Lower limb muscle volume	Leg press Chest press Knee extension	–
Bjørnsen (2016) [32], Norway	I: 17, 69 ± 7, male C: 17, 67 ± 5, male	Free weight exercises, 3 times a week	500 mg vitamin C and 117.5 mg vitamin E, twice a day	Placebo: cellulose and dicalsium phosphate	12	Lean body mass Muscle thickness	Biceps curl Knee extension Leg press	–
Bobeuf (2011) [33], Canada	I: 14, 64.3 ± 3.8, mixed (50%) C: 17, 67 ± 3.7, mixed (52.9%)	Resistance training, 60 min sessions, 3 times a week	1000 mg vitamin C ascorbate and vitamin E, once a day	Placebo: 100 mg lactose	24	ASMM Muscle mass	–	–
Brose (2003) [34], Canada	I: 14, 69.6 ± 5.4, mixed (42.8%) C: 14, 69.1 ± 4.8, mixed (50%)	Resistance training using machines, 3 times a week	5 g creatine and 2 g dextrose, once a day	Placebo: 7 g dextrose	14	Fat free mass	Grip strength Knee extension Leg press	–
Chrusch (2001) [35], Canada	I:16, 70.4 ± 6.4, male C:14, 71.1 ± 6.7, male	Resistance training using machines, 3 times a week	Creatine supplement: 0.3 g/kg/day for the first 5 days (loading phase) and 0.07 g/kg/day thereafter, once a day, for 11 weeks	Placebo: sucrose-flour mixture	12	Lean body mass	Bench press Knee extension Leg press	–
Cornish (2018) [36], Canada	I:11, 71.4 ± 6.2, male C:12, 70.9 ± 5, male	Resistance training, 60 min sessions, 3 times a week	3.0 g omega-3 fatty acid combined 1.98 g EPA^i^ and 0.99 g DHA^j^, once a day	Placebo: 3.0 g omega 3–6-9 blend	12	Body mass Lean body mass	Chest press Leg press	TUG 6-min walk
Da Boit (2017) [37], UK	I: 27, 70.1 ± 4, mixed (48.1%) C: 23, 70.9 ± 4.2, mixed (43.4%)	Lower body resistance training, twice a week	3.0 g omega–3 fatty acids containing 2.1 g EPA and 0.6 g DHA, once a day	Placebo: 3.0 g safflower oil	18	Muscle anatomic cross-sectional area	Knee extension	Chair stand SPPB^k^ 4 m walk
Edholom (2017) [38], Sweden	I: 20, 67.2 ± 1.3, female C: 17, 67.9 ± 2.1, female	Resistance training, 60 min sessions, twice a week	Healthy diet: following a dietary consultation and a diet plan with the current dietary guidelines in Europe and US	Usual diet	24	Lean body mass	Knee extension Leg press	Chair stand Single leg stance Squat jump TUG
Holwerda (2018) [39], Netherlands	I: 21, 69 ± 4.6, male C: 20, 71 ± 4.5, male	Resistance exercise training, 3 times a week	21 g leucine-enriched whey protein (3 g total leucine), once a day	Placebo	12	ASMM BMI Body mass Lean body mass	Knee extension Leg press	Chair stand SPPB 4 m walk
Kawada (2013) [40], Japan	Ia: 10, 65 ± 1, mixed (50%) Ib: 11, 67 ± 3, mixed (72.3%) C: 8, 70 ± 1, mixed (50%)	Low-intensity resistance training, twice a week	Ia: 3.0 g essential amino acid supplements with milk, twice a day Ib: 6.0 g essential amino acid supplements with milk, twice a day	Placebo: 3 g dextrin-contained powder with milk, once a day	24	Cross sectional area of Psoas major muscle	–	Gait speed Obstacle course walk 6-min walk
Leenders (2013) [41], Netherlands	I: 27,70.9 ± 5.4, mixed (44.4%) C: 26, 69.5 ± 3.6, mixed (46.2%)	Resistance training, 3 times a week	15 g milk protein, once a day	Placebo: 7.13 g lactose and 0.42 g calcium only	24	BMI Lean body mass Lower limb lean mass	Grip strength Leg press	Chair stand
Mori (2018) [42], Japan	I: 25, 70.6 ± 4.2, female C: 25, 70.6 ± 4.2, female	Resistance training, twice a week	25 g leucine enriched whey protein, once a day	Usual diet	24	BMI Lower limb lean mass SMI^l^ Upper limb lean mass	Grip strength Knee extension	Gait speed
Nabuco (2018) [43], Brazil	Ia: 23, 66.2 ± 9.4, female Ib: 24, 67.5 ± 5.2, female C: 23, 66.5 ± 7.2, female	Resistance training, 3 times a week	Ia: 27.1 g whey protein after resistance training, 3 times a week Ib: 27.1 g whey protein before resistance training, 3 times a week	Placebo: maltodextrin drink	12	Lower limb lean mass Skeletal muscle mass Upper limb lean mass	Biceps curl Chest press Knee extension	Chair stand Gait speed
Nagai (2019) [44], Japan	I: 17, 72.7 ± 1.4, mixed (64.7%) C: 19, 73.5 ± 2.3, mixed (68.4%)	Latex band training, squat, and tai chi, 90 min sessions, once a week	60 mg maslinic acid, once a day	Placebo: jelly without maslinic acid	12	BMI Body mass Fat free mass Skeletal muscle mass Segmental muscle mass	Grip strength	Chair stand Gait speed
Nakayama (2020) [45], Japan	I: 61, 71.4 ± 6.2, mixed (74%) C: 61, 70.4 ± 5.5, mixed (77%)	Body weight exercises and 5 medicine ball exercises, daily	Low-dose milk protein: 10.1 g protein, once a day	Placebo: Isocaloric carbohydrate	24	Body mass Lean body mass	Grip strength Knee extension Knee flexion Push up	Chair stand Gait speed TUG
Nilsson (2020) [46], Canada	I: 16, 77.4 ± 11.2, male C: 16, 74.4 ± 5.2, male	Home based resistance training with elastic bands, 3 times a week	Multi nutrients: 24 g whey protein, 16 g micellar casein contained 416 mg calcium, 3 g creatine, vitamin D 1000 IU, and omega-3 fish-oil containing 1.51 g EPA and 0.95 g DHA, once a day	Placebo: collagen and sunflower oil	12	ASMM Body mass BMI Lean body mass	Grip strength Knee extension Leg press	Chair stand SPPB Stair climb TUG 4 m walk
Pinto (2016) [47], Brazil	I: 13, 67.4 ± 4.7, mixed (*unknown*) C: 14, 67.1 ± 6.3, mixed (*unknown*)	Resistance training, 3 times a week	5 g Creatine monohydrate, once a day	Placebo: 5 g maltodextrin	12	Body mass Lean body mass SMI	Bench press Leg press	–
Seino (2018) [48], Japan	I: 40, 73.4 ± 4.3, mixed (85%) C: 40, 73.7 ± 4.3, mixed (82.5%)	Weight-bearing exercise and exercises using a resistance band and Pilates ball, 60 min sessions, twice a week	Fortified milk containing 10.5 g total milk protein, 3.9 g fat, 9.3 g carbohydrate, and 337 mg calcium at lunch and micronutrient beverage at breakfast, daily	Usual diet	12	Body mass Lean body mass Lower limb lean mass SMI	Grip strength Knee extension	Chair stand Gait speed One leg standing with eyes open TUG
Stout (2013) [49], USA	I: 24, 73 ± 4.9, mixed (54.2%) C: 24, 73 ± 4.9, mixed (54.2%)	Resistance exercise, 3 times a week for 21 weeks	1.5 g calcium and 4 g carbohydrate, twice a day	Placebo: 200 mg calcium and 4 g carbohydrate	24	Lean body mass Lower limb lean mass Upper limb lean mass	Grip strength	Chair stand
Sugihara Junior (2018) [50], Brazil	I: 15, 67.4 ± 4.1, female C: 16, 67.8 ± 4.1, female	Resistance training using a combination of free weights and machines, 45 ~ 50 min sessions, 3 times a week	35 g whey protein, immediately after each resistance training	Placebo: 35 g maltodextrin	12	Body mass BMI Lower limb lean mass SMI Upper limb lean mass	Biceps curl Chest press Knee extension	–
Tarnopolsky (2007) [51], Canada	I: 21,70.7 ± 4.5, mixed (47.6%) C: 18, 71.1 ± 5.5, mixed (55.6%)	Resistance exercise using machines, twice a week	5 g creatine monohydrate and 6 g conjugated linoleic acid, once a day	Placebo: dextrose and safflower oil	24	Body mass BMI Fat free mass	Biceps curl Chest press Knee extension Leg press	Chair stand Gait speed Stair climb Standing balance
Villanueva (2014) [52], USA	I: 7, 68.7 ± 6.8, male C: 7, 68.7 ± 6.6, male	Progressive overload, total body periodized resistance program, 3 times a week	First 5 days: 0.1 g/kg body weight of creatine 3 times a day and 35 g whey protein once a day Following days: 0.07 g/kg body weight of creatine and 35 g whey protein, once a day	Usual diet	12	Lean body mass	Bench press Leg press	Stair climb 400 m walk

^a^The resistance exercise of the control group applied in the same manner as in the experimental group

^b^ The duration of intervention in most studies were the same as the follow-up period

^c^ I, intervention group ^d^ C, control group ^e^ IU, international unit ^f^ BMI, body mass index ^g^ *ASMM,* appendicular skeletal muscle mass, ^h^ TUG, timed up and go ^i^ EPA, eicosapentaenoic acid ^j^ DHA, docosahexaenoic acid ^k^ SPPB, short physical performance battery ^l^ SMI, skeletal muscle mass index

All of the studies administered supervised exercise programs except one study [46] which included home-based resistance training with consistent encouragement. In almost all the studies, the exercise programs were performed twice (6 studies) or three times (16 studies) a week on nonconsecutive days; the exercise programs were performed daily in two studies and once a week in one study.

The RCTs provided protein (eight studies), creatine (five studies), long chain n-3 polyunsaturated fatty acids (PUFA omega-3) (two studies), calcium (one study), maslinic acid (one study), vitamins C and E (two studies), creatine and linoleic acid (one study), creatine and protein (one study), vitamin D (one study), and multinutrients containing more than three nutrients (three studies). Most studies provided nutritional supplements in pill, capsule, powder or drink forms, and a study provided a personalized and nutritionally balanced diet [38]. Most studies provided control groups with an isocaloric placebo. Two studies provided the control groups with pills or capsules containing some nutrients, such as calcium or omega-3 [36, 49]. The intervention period ranged between 8 and 24 weeks: eight weeks in one study, 12 weeks in 13 studies, 14 weeks in one study, 18 weeks in one study, and 24 weeks in nine studies.

### Risk of Bias

**Fig. 2:**
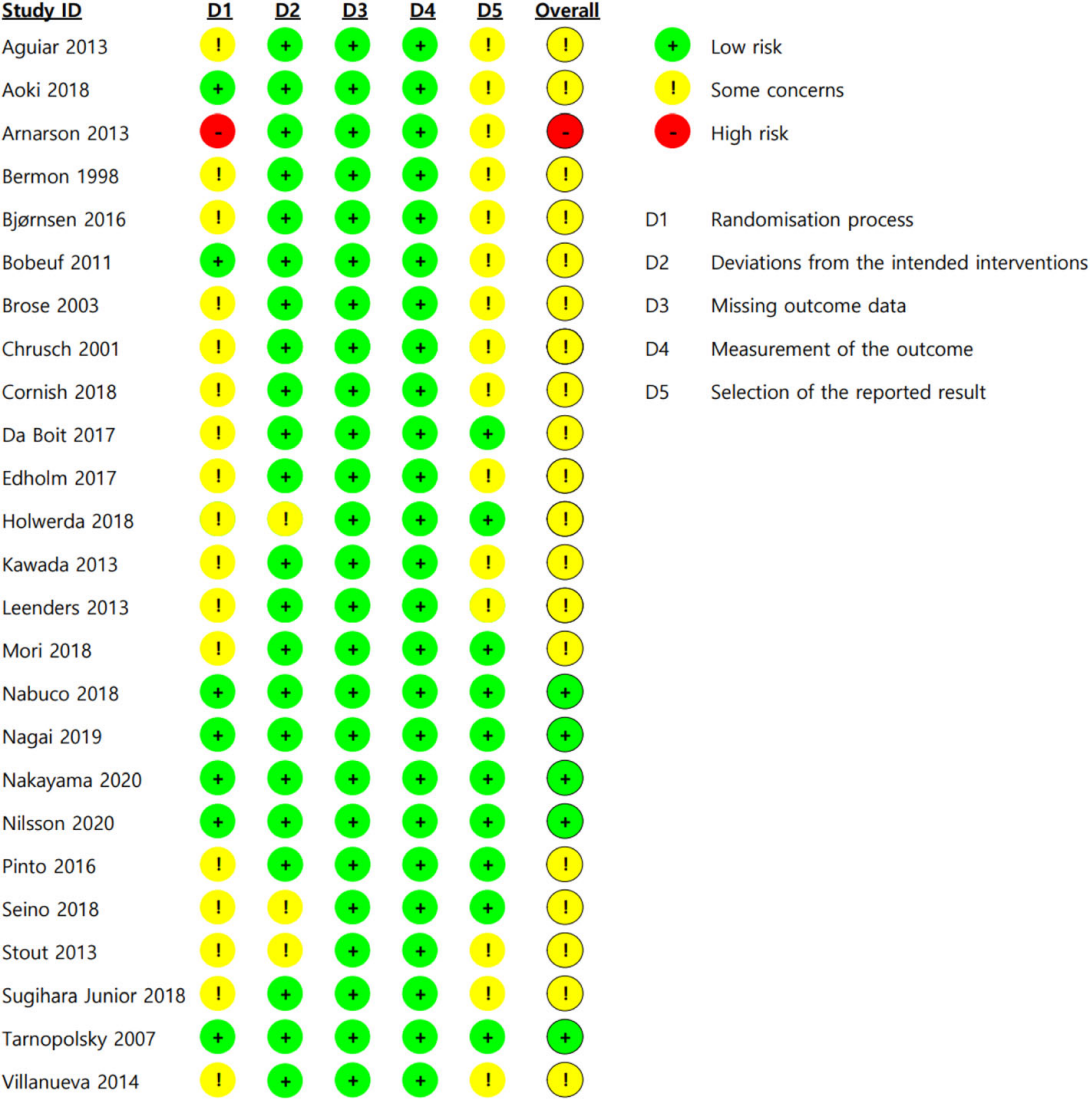
Risk of bias of the included studies

The risk of bias results for the 25 RCTs are demonstrated in Fig. 2. Regarding the randomization process, seven studies had a low risk of bias, 17 had some concerns, and one study had a high risk of bias because of a failure to conceal group allocation. Regarding deviation from the intended intervention, three had some concerns, and the others had a low risk of bias. As there were no studies in which missing values were judged to have an impact on the study results, all studies had a low risk of bias in the domain of missing outcome data. All studies had a low risk of bias in the domain of measurement of outcome, either because the outcome assessor was blinded or the outcome assessor’s awareness of the group assignments was judged to not affect the measurement of muscle mass, strength, or physical function. In the fifth domain, the selection of the reported results, 10 studies had a low risk of bias, while the other 15 studies had some concerns because of the absence of a prespecified trial protocol. Overall, five RCTs had a low risk of bias, 19 RCTs had some concerns, and one study had a high risk of bias.

### Effects of resistance training and nutritional interventions compared with those of resistance training only on muscle mass, muscle strength, and physical functional performance

**Fig. 3:**
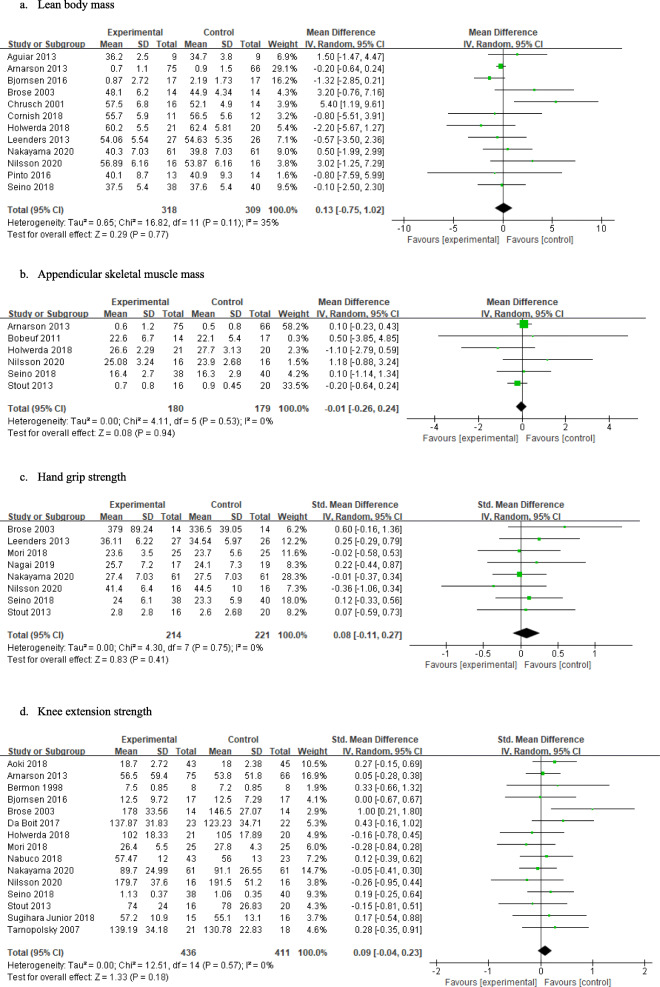
Effects of resistance training and nutritional interventions compared with those of resistance training only on muscle mass, muscle strength, and physical functional performance

The effect sizes and 95% confidence intervals (95% CIs) for individual studies and all studies are shown in Fig. 3. The results of the meta-analysis showed no significant effects on lean body mass (*n* = 12, MD 0.13, 95% CI − 0.75 to 1.02), appendicular skeletal muscle mass (*n* = 6, MD -0.01, 95% CI − 0.26 to 0.24), hand grip strength (*n* = 8, SMD 0.08, 95% CI − 0.11 to 0.27), knee extension strength (*n* = 15, SMD 0.09, 95% CI − 0.04 to 0.23), the chair stand test results (*n* = 7, MD -0.13, 95% CI − 0.43 to 0.17), or the timed up-and-go test results (*n* = 6, MD 0.02, 95% CI − 0.16 to 0.20). The I^2^ values for all outcomes except lean body mass were zero, indicating that heterogeneity might not be important for these outcomes and that lean body mass might represent moderate heterogeneity (I^2^ = 35%).

### Subgroup analysis according to type of nutritional interventions

The results of the subgroup analyses according to the type of nutritional interventions are shown in Table 2. The subgroup analyses for lean body mass showed significant differences between the types of nutritional interventions (Chi^2^ = 7.28, *p* = .03). Among the nutritional interventions, only those with creatine showed significant effects on lean body mass (*n* = 4, MD 2.61, 95% CI 0.51 to 4.72). Regarding the other subgroup analyses, there were no significant differences in appendicular skeletal muscle mass (χ^2^ = 0.62, *p* = .43), hand grip strength (χ^2^ = 0.12, *p* = .73), knee extension strength (χ^2^ = 4.89, *p* = .09), chair stand test results (χ^2^ = 1.05, *p* = .43), or timed up-and-go test results (χ^2^ = 1.02, *p* = .31). Although creatine showed significant effects on knee extension strength (*n* = 2, SMD 0.74, 95% CI 0.09 to 1.38), there were no significant subgroup differences.

**Table 2:** Subgroup analysis: effects by type of nutritional interventions on muscle mass, muscle Strength, and physical functional performance

Subgroups	Lean body mass					Appendicular skeletal muscle mass				
	n	MD	95% CI	I^2^	Subgroup differences	n	MD	95% CI	I^2^	Subgroup differences
Creatine	4	2.61	0.51 ~ 4.72	9%	χ^2^ = 7.28 (*p* = .03)	–	–	–	–	χ^2^ = 0.62 (*p* = .43)
Multi-nutrients	2	0.94	−1.94 ~ 3.82	36%		2	0.39	−0.68 ~ 1.45	0%	
Protein	4	−0.21	− 0.57 ~ 0.14	0%		2	−0.20	−1.23 ~ 0.82	47%	
Subgroups	Hand grip strength					Knee extension strength				
	n	SMD	95% CI	I^2^	Subgroup differences	n	SMD	95% CI	I^2^	Subgroup differences
Creatine	–	–	–	–	χ^2^ = 0.12 (*p* = .73)	2	0.74	0.09 ~ 1.38	7%	χ^2^ = 4.89 (*p* = .09)
Multi-nutrients	2	−0.04	−0.48 ~ 0.40	21%		2	0.05	−0.36 ~ 0.46	12%	
Protein	3	0.05	−0.21 ~ 0.31	0%		6	−0.02	−0.20 ~ 0.17	0%	
Subgroups	Chair stand test					Timed up and go test				
	n	MD	95% CI	I^2^	Subgroup differences	n	MD	95% CI	I^2^	Subgroup differences
Multi-nutrients	2	0.15	−0.41 ~ 0.72	0%	χ^2^ = 1.05 (*p* = .31)	2	0.26	−0.31 ~ 0.83	32%	χ^2^ = 1.02 (*p* = .31)
Protein	3	−0.21	−0.60 ~ 0.19	0%		2	−0.06	−0.28 ~ 0.17	0%	

### Moderator analysis with Meta-regression

As recommended [27], meta-regression was conducted when there were more than ten studies in a meta-analysis; therefore, meta-regression was performed on lean body mass (*n* = 12) and knee extension strength (*n* = 15). The mean age of the subjects and duration of interventions were included as explanatory variables in a univariate regression model. In the meta-regression, neither the mean age of the subjects (β = 0.01, SE = 0.04, t = 0.18, *p* = .858) nor the duration of interventions (β = − 0.001, SE = 0.02, t = − 0.04, *p* = .965) were associated with lean body mass. These moderators were not contributing variables to knee extension strength (β = − 0.03, SE = 0.03, t = − 0.90, *p* = .383; β = − 0.01, SE = 0.01, t = − 0.45, *p* = .660, respectively).

### Sensitivity analysis

Sensitivity analysis was conducted to compare the effect sizes, 95% CIs, and I^2^ values by excluding two studies that provided some nutrients to control groups. There were no significant differences in lean body mass (*n* = 11, MD 0.20, 95% CI − 0.74 to 1.14), appendicular skeletal muscle mass (*n* = 5, MD 0.09, 95% CI − 0.23 to 0.40), hand grip strength (*n* = 7, MD 0.08, 95% CI − 0.12 to 0.28), knee extension strength (*n* = 14, MD 0.10, 95% CI − 0.04 to 0.24), chair stand test results (n = 7, MD -0.13, 95% CI − 0.43 to 0.17), or timed up-and-go test results (*n* = 4, MD 0.01, 95% CI − 0.18 to 0.20). The I^2^ values for all outcomes except for lean body mass were zero, indicating that heterogeneity might not be important for these outcomes and that lean body mass might have moderate heterogeneity (I^2^ = 40%).

### Certainty of evidence

There were six outcomes to compare the quality of evidence for resistance training combined a nutritional intervention compared to resistance training alone for muscle function. The hand grip strength and chair stand test were of moderate certainty. The other outcomes were low. The GRADE summary of the findings is shown in Additional file 2.

## Discussion

Nutrient-dense foods that ensure sufficient intake of energy, protein and micronutrients are important to prevent frailty and sarcopenia and promote physical activity. However, to date, the optimal type of nutritional intervention or supplementation for the prevention of frailty and sarcopenia is unclear. This study was conducted to compare the synergistic effect of nutritional interventions combined with resistance training with that of resistance training alone. This study can provide insight into resource optimization and strategies to prevent frailty and sarcopenia.

This systematic review and meta-analysis showed that there were no additional effects of nutritional interventions when combined with resistance training on muscle mass, strength, or physical function. Of note, in two studies, the control conditions included some nutrients that have biological benefits [36, 49], which likely reduced the calculated effect size when the data for the control conditions were pooled. However, the findings of the sensitivity analysis showed little possibility of blunted effects. One of the possible reasons for this lack of significant results is that the analysis included studies of healthy older adults who might not have nutrient deficiencies with the usual diets [23]. Healthy diets provide a broad range of micronutrients and bioactive nonnutrients as well as macronutrients that might not be included in the experimental supplements in trials. In addition, since diets are patterned, isolating the effects of individual experimental supplements might not be possible without controlling for the usual diet. Thus, the effects of nutritional interventions might be blunted among older adults who habitually consume sufficient nutrients. However, in previous studies that provided vitamin D-deficient and mobility-limited older adults with a protein mixture containing 20 g protein, 800 IU vitamin D, 350 mg calcium, and other minerals once a day for 6 months with an exercise program, there were no differences in muscle function parameters such as leg strength, gait speed, and short physical performance battery between this group and the exercise-only control group except in muscle density [53, 54]. In another study that also provided sarcopenic older adults who had low protein intake with multinutrient supplements containing 21 g protein, 800 IU vitamin D and other nutrients once a day for 3 months with an exercise program, there were no differences between the two groups, although both groups exhibited improved muscle function [55]. It is necessary to additionally consider the dose of the nutrient and duration of intervention and monitor dietary energy intake. Despite the lack of evidence, greater benefits of resistance training along with nutritional supplementation are expected in older adults who already have poor muscle function or habitually have low nutrient intake.

In the subgroup analysis of the types of nutrients, only creatine showed significant effects on lean body mass. Among the five studies included in this meta-analysis, four administered 5 g creatine daily combined with resistance training 3 times a week for 12 weeks [28, 34, 47]  or twice a week for 24 weeks [51], and one administered 5 g creatine four times a day for the first 5 days of the loading phase and 3 g creatine daily for the maintenance phase combined with resistance training 3 times a week for 7 weeks [31]. Recent systematic reviews similarly identified the additive effect of creatine during resistance training on body composition, muscle strength, and physical function [56, 57]. As skeletal muscle has no capacity for creatine biosynthesis, the consumption of creatine-containing food or supplementation of creatine increases creatine and phosphocreatine levels in skeletal muscle and elevates phosphate resynthesis (energy buffer) during high-energy demanded exercise, such as repetitive resistance training training [58, 59, 60]. Creatine helps to increase muscle mass and strength by indirectly increasing work capacity, and the combination of creatine supplementation and resistance training promotes muscle protein synthesis. Alternatively, creatine supplementation may enhance muscle protein synthesis stimulating signaling pathways (myogenic regulatory factors), which facilitate myosatellite cell proliferation and differentiation [61]. Controversy exists as to whether creatine stores and metabolism are affected by aging, but creatine supplements can account for dietary changes and reductions in physical activity with aging [57]. The effect sizes for variables other than lean body mass were not significant in this study. Additional meta-analyses including more experimental studies are needed to verify the effects of creatine on muscle mass and function in older adults.

As proteins provide amino acids that are essential for muscle protein synthesis and act as anabolic stimuli, protein consumption increases muscle mass, and protein consumption following resistance training enhances net protein utilization, attenuating exercise-induced muscle protein breakdown [60, 62]. The combination of protein supplements and exercise was expected to have a synergistic effect on muscle function, but the findings of this study did not support this hypothesis. On the other hand, in a previous meta-analysis, protein supplements for sarcopenic older adults along with exercise showed a larger effect size than exercise alone and no intervention [24]. The previous meta-analysis was conducted in frail, sarcopenic, or mobility-limited older adults and included not only community-dwelling older adults but also institutionalized older adults. Individuals with existing nutritional deficiencies or poor muscle function might have been shown to respond better to accompanying nutritional supplements than to exercise alone. Additional studies are needed to determine whether the inconsistency in findings resulted from the characteristics of the subjects.

Muscle protein synthesis through protein intake in older adults should be maximized with consideration of the frequency, distribution, and other nutritional components, such as creatine, vitamins, and fatty acids [22, 60]. It is recommended that older adults consume ≥0.4 g/kg per meal and 1.2–1.6 g/kg per day to induce muscle protein synthesis saturation to thus support muscle function [60]. Among the included studies, most studies provided 10.1 g–25 g protein once a day [39, 41, 42, 45]  or 20 g ~ 35 g protein 3 times a week on the days exercise was performed [30, 43, 50], which could not take into account frequency and distribution. A previous review showed that multi-ingredient protein supplements have the potential to increase the benefits of resistance training, but there were no differences in the effects on muscle mass and strength between multi-ingredient protein and single protein [63]. The impact of multiple nutrients is unclear, but there are complex interactions between food components inducing potential synergistic effects. Thus, nutritional interventions involving dietary modifications with various and balanced nutrients or whole food approaches rather than a single specific nutrient can be effective in improving muscle mass and function [64]. Among the 25 RCTs, three provided multinutrients that were arbitrarily defined as containing three or more nutrients. Of the three studies, only one used a whole-diet approach. The number of studies was too small to verify the effect of the whole-food or whole-diet approach.

Nutritional effects may not manifest following dietary interventions of short durations. Although the meta-regression results did not show that the intervention period was associated with effect sizes, a 6-year longitudinal study showed a positive relationship between daily protein intake and muscle strength [65]; nutritional contributions can be expected to be observed in the long term. Thus, despite the nonsignificant results, nutritional interventions may still be beneficial for older adults who do not lack nutrients. With aging, muscle loss (breakdown) occurs more rapidly than muscle synthesis, so additional supplements may be required. In addition, older adults experience declines in food intake because of changes in appetite and a lack of hunger, which is referred to as ‘anorexia of aging’ [66]. As consumed food is metabolized to synthesize energy for organ function, poor nourishment leads to body fat and muscle being catabolized to provide energy. Not only a lack of specific nutrients but also the consumption of an insufficient amount of food contributes to weight loss and declines in muscle mass, strength and physical function, which can lead to physical frailty and sarcopenia. Thus, the consumption of an adequate amount of food containing nutrients essential for muscle function is important to maintain muscle mass, strength, and physical function [22, 58]. Considering that changes occur in various physiological functions as well as muscle function, interventions with a balanced diet are important in older people. As nutritional interventions have the advantages of low costs and high availability and accessibility, additional studies are necessary to determine whether they can be effective in preventing frailty and sarcopenia.

This study has several limitations. First, this meta-analysis included only retrievable RCTs that were published in English, which may have contributed to language bias. Second, this study in healthy older adults might not have demonstrated significant effects on muscle mass, muscle strength, and physical function due to the ceiling effect. Additional systematic reviews and meta-analyses are needed to identify the additional effects of nutritional interventions when combined with resistance training among dynapenic, sarcopenic, or frail older adults. Third, the range of nutritional interventions included in this study were vast because each nutrient has a different mechanism that affects muscle synthesis, function, or prevention of muscle damage. Future studies need to be more focused on a specific nutritional intervention. Finally, as mentioned above, the amount, frequency, and distribution of nutrients administered are important to consider to fully assess the effects of nutritional interventions; however, these factors were not assessed in the meta-analysis.

As the levels of variability in muscle mass and functional measurements are quite high in older adults, it is hard to obtain adequate statistical power to verify differences between groups in many studies on nutritional interventions. This meta-analysis showed that nutritional interventions have no additional effect on body composition, muscle strength, or physical function when combined with resistance training. Only creatine showed synergistic effects with resistance training on muscle mass. The enhanced effect of nutritional interventions for unhealthy older adults, such as frail, sarcopenic, or nutritionally deficient older adults, needs to be investigated in future studies. The long-term effects of nutrition on muscle function also need to be studied. In addition, additional studies should be conducted to identify the dietary parameters that maximize nutritional effects on muscle protein synthesis, including dose, frequency, distribution, and recipes that take into account interactions with other nutrients. Health-promoting interventions such as exercise and diet are important for at-risk older adults to prevent clinically evident disability. This systematic review and meta-analysis provides a comprehensive synthesis of the experimental results available to date for health practitioners and researchers to establish intervention strategies or public health policies.

## Supplementary Information

**Additional file 1.** PRISMA checklist.

**Additional file 2.** Summary of findings and analysis of the quality of evidence.

## Data Availability

The authors can confirm that all relevant data are included in the article.

## Abbreviations

CI: Confidence interval
GRADE: Grading of recommendations, assessment, development and evaluation
IU: International unit
MD: Mean difference
PRISMA: Preferred reporting items for systematic reviews and meta-analyses
PUFA: Polyunsaturated fatty acid
RCT: Randomized controlled trial
RoB: Risk of bias
SD: Standard deviation
SMD: Standardized mean difference.
UK: United Kingdom
USA: United States of America
