# Correction: Does the combination of resistance training and a nutritional intervention have a synergic effect on muscle mass, strength, and physical function in older adults? A systematic review and meta-analysis

**DOI:** 10.1186/s12877-022-03110-7

**Published:** 2022-06-28

**Authors:** MoonKi Choi, Hayeon Kim, Juyeon Bae

**Affiliations:** 1 College of Nursing, Kangwon National University, Chuncheon-si, Gangwon-do 24341 Republic of Korea; 2 Seoul Women’s College of Nursing, Ganhodae-ro 38, Seodaemun-gu, Seoul, 03617 Republic of Korea; 3 Department of Nursing, Yeoju Institute of Technology, Sejong-ro 338, Yeoju-si, Gyeonggi-do 12652 Republic of Korea

**Correction: BMC Geriatr 21, 639 (2021)**

**https://doi.org/10.1186/s12877-021-02491-5**

After publication of this article [1], the authors reported that in this article Figs. 3e and 3f were missing from this article; the figure should have appeared as shown below.

**Fig. 3:**
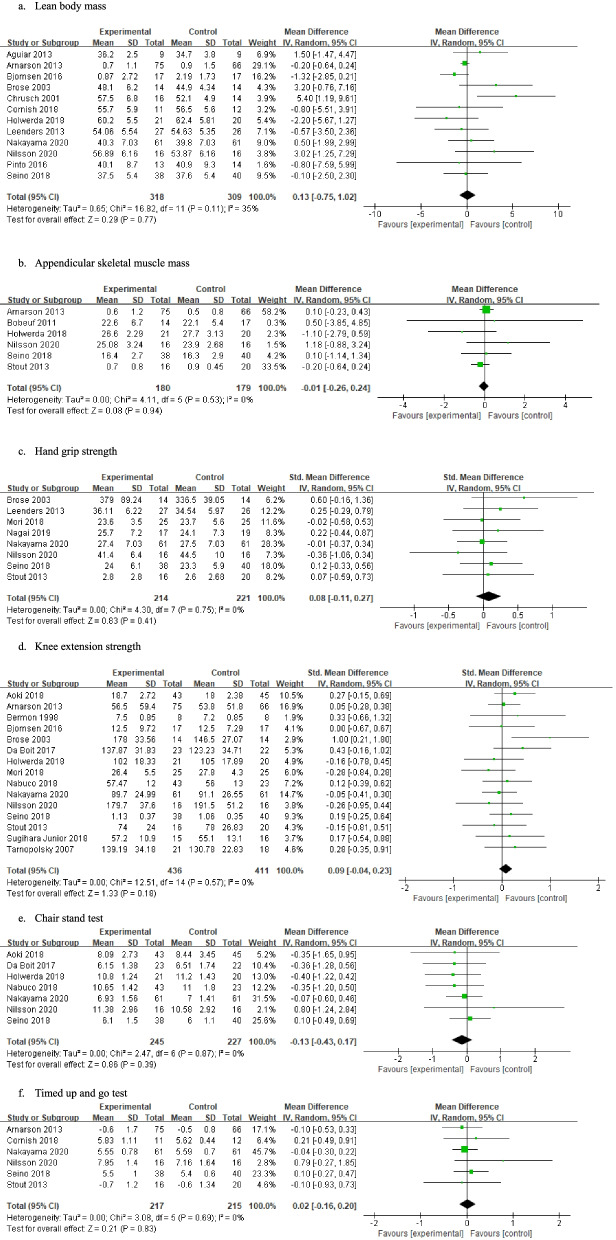
Effects of resistance training and nutritional interventions compared with those of resistance training only on muscle mass, muscle strength, and physical functional performance

The original article [1] has been updated.
