# Supplementary material for: Does the combination of resistance training and a nutritional intervention have a synergic effect on muscle mass, strength, and physical function in older adults? A systematic review and meta-analysis
Source: BMC Geriatr. 2021 Nov 12;21:639. doi: 10.1186/s12877-021-02491-5 (PMC8588667; doi:10.1186/s12877-021-02491-5)
Supplement: Supplementary file 2 — Additional file 2. Summary of findings and analysis of the quality of evidence. [file 12877_2021_2491_MOESM2_ESM.docx]

**Additional file 2 Summary of findings and analysis of the quality of evidence**

| **Certainty assessment** | | | | | | | | **Summary of findings** | | | |
| --- | --- | --- | --- | --- | --- | --- | --- | --- | --- | --- | --- |
| Outcome | No of Participants  (studies) | Risk of bias | Inconsistency | Indirectness | Imprecision | Publication bias | Overall certainty of evidence | With resistance training only | With resistance training combined diet | Relative effect (95% CI) | Absolute effect |
| Lean body mass | 627 (12 RCTs) | serious ^a^ | not serious | not serious | serious ^b^ | none | ⨁⨁◯◯ LOW | 309 | 318 | - | MD 0.13 higher (0.75 lower to 1.02 higher) |
| Appendicular skeletal muscle mass | 359 (6 RCTs) | serious ^c^ | not serious | not serious | serious ^b^ | none | ⨁⨁◯◯ LOW | 179 | 180 | - | MD 0.01 lower (0.26 lower to 0.24 higher) |
| Hand grip strength | 435 (8 RCTs) | not serious | not serious | not serious | serious ^b^ | none | ⨁⨁⨁◯ MODERATE | 221 | 214 | - | SMD 0.08 higher (0.11 lower to 0.27 higher) |
| Knee extension strength | 847 (15 RCTs) | serious ^d^ | not serious | not serious | serious ^b^ | none | ⨁⨁◯◯ LOW | 411 | 436 | - | SMD 0.09 higher (0.04 lower to 0.23 higher) |
| Chair stand test | 472 (7 RCTs) | not serious | not serious | not serious | serious ^b^ | none | ⨁⨁⨁◯ MODERATE | 227 | 245 | - | MD 0.13 lower (0.43 lower to 0.17 higher) |
| Timed up and go test | 432 (6 RCTs) | serious ^c^ | not serious | not serious | serious ^b^ | none | ⨁⨁◯◯ LOW | 215 | 217 | - | MD 0.02 higher (0.16 lower to 0.2 higher) |

CI=Confidence interval; RCT=Randomized controlled trial; MD= Mean difference; SMD= Standardized mean difference; GRADE Working Group grades of evidence; High certainty = very confident that the true effect lies close to that of the estimate of the effect; Moderate certainty = moderately confident that the true effect lies close to that of the estimate of the effect;

Low certainty = limited confidence in the effect estimate, the true effect may be substantially different from the estimate of effect; Very Low certainty = very little confidence in the effect estimate, the true effect is likely to be substantially different from the estimate of effect

Explanations

a. Downgraded by one level due to risk of bias; Nine studies did not report the randomization process and one study failed to conceal group allocation.

b. Downgraded by one level due to imprecision; 95% CIs around the effects were wide.

c. Downgraded by one level due to risk of bias; Three studies did not report the randomization process and one study failed to conceal group allocation.

d. Downgraded by one level due to risk of bias; Eight studies did not report the randomization process and one study failed to conceal group allocation.
